# A comprehensive overview on antibody-drug conjugates: from the conceptualization to cancer therapy

**DOI:** 10.3389/fphar.2023.1274088

**Published:** 2023-09-18

**Authors:** Federico Riccardi, Michele Dal Bo, Paolo Macor, Giuseppe Toffoli

**Affiliations:** ^1^ Experimental and Clinical Pharmacology Unit, Centro di Riferimento Oncologico (CRO), IRCCS, Aviano, Italy; ^2^ Department of Life Sciences, University of Trieste, Trieste, Italy

**Keywords:** antibody-drug conjugate, antibodies, linkers, payloads, target therapy, cancer treatment

## Abstract

Antibody-Drug Conjugates (ADCs) represent an innovative class of potent anti-cancer compounds that are widely used in the treatment of hematologic malignancies and solid tumors. Unlike conventional chemotherapeutic drug-based therapies, that are mainly associated with modest specificity and therapeutic benefit, the three key components that form an ADC (a monoclonal antibody bound to a cytotoxic drug via a chemical linker moiety) achieve remarkable improvement in terms of targeted killing of cancer cells and, while sparing healthy tissues, a reduction in systemic side effects caused by off-tumor toxicity. Based on their beneficial mechanism of action, 15 ADCs have been approved to date by the market approval by the Food and Drug Administration (FDA), the European Medicines Agency (EMA) and/or other international governmental agencies for use in clinical oncology, and hundreds are undergoing evaluation in the preclinical and clinical phases. Here, our aim is to provide a comprehensive overview of the key features revolving around ADC therapeutic strategy including their structural and targeting properties, mechanism of action, the role of the tumor microenvironment and review the approved ADCs in clinical oncology, providing discussion regarding their toxicity profile, clinical manifestations and use in novel combination therapies. Finally, we briefly review ADCs in other pathological contexts and provide key information regarding ADC manufacturing and analytical characterization.

## 1 Introduction

Cancer is a multi-stage progression that transforms normal healthy cells into malignant lesions (Cooper, 2000). It is the leading cause of death worldwide, accounting for nearly 10 million deaths in 2020, and one of the most challenging diseases to treat (Sung et al., 2021). Conventional therapeutic strategies are grouped into chemotherapy, radiotherapy, immunotherapy and surgery, with the former being the main approach for treating various cancers (Dan et al., 2018). Although most remarkable goals have been achieved with small cytotoxic drugs, they had several drawbacks that limited their efficacy, including a low therapeutic index and a high off-tumor effect (Senapati et al., 2018). Usually, the low effectiveness of chemotherapy provoked a high incidence of severe side effects in patients, mainly caused by the non-specific action of chemotherapeutic drugs on rapidly dividing normal cells (Senapati et al., 2018). Therefore, one of the hot topics in the field concerns the discovery of new chemical agents with enhanced therapeutic efficacy and that preferentially ablate tumor-derived cells, without harming the body itself (Casi and Neri, 2012). In the field of immunotherapy, several monoclonal antibodies (mAbs) have been clinically approved as they showed therapeutic benefits in both hematologic malignancies and solid tumors by selectively targeting cancer cells and by activating direct and/or indirect killing mechanisms (Weiner et al., 2009; Kimiz-Gebologlu et al., 2018; Castelli et al., 2019). However, issues including tissue accessibility, poor pharmacokinetics and lame interactions with the immune system have led to the need to exploit newer, safer and more effective targeted therapies (Chames et al., 2009; Talotta et al., 2019). In 1907, German nobel laureate Paul Erlich postulated that there could exist compounds that would selectively target pathogenic microbes, such as bacteria, while sparing normal cells, a concept gone down in history as “zauberkugel” (magic bullet) (Schwartz, 2004). From his hypothesis, an innovative therapeutic approach, known as Antibody-Drug Conjugate (ADC), has arisen in the oncology field and was firstly 40 used years ago to treat patients with advanced cancer (Ford et al., 1983). ADCs are an emerging class of pharmacological compounds that combine the potency of anti-cancer drugs (often called payloads) with the specificity of mAbs to the tumor site, thus combining chemotherapy and immunotherapy. They are composed of three portions, a mAb, an organic spacer and a cytotoxic drug. Ideally, they use the mAb targeting ability to take the cytotoxic agent, which is bound to the mAb via a stable linker, to the cancerous cells or to the cellular components of the tumor microenvironment (TME) where it can exert its anti-tumor activity and lead to cell death (Baah et al., 2021; Mckertish and Kayser, 2021; Fu et al., 2022). Compared with standard chemotherapy, this strategy offers several advantages including better drug tolerability, cytotoxicity even at low concentrations, drug stability in the bloodstream and in lysosomes, reduced off-target effects, and systemic toxicity, all features that contribute to the expansion of its therapeutic potential (Khongorzul et al., 2020; Drago et al., 2021; Mckertish and Kayser, 2021; Fu et al., 2022). Beginning with the first ADC, which was approved by the Food and Drug Administration (FDA) in 2000 (Norsworthy et al., 2018), and given the ever-evolving technology of mAbs, linkers, and payloads, by April 2023 13 different ADCs have been FDA-approved for clinical use for both solid and hematologic malignancies, setting the stage for a new era of targeted cancer therapy (Dumontet et al., 2023). In addition, a few studies have already addressed the potential use of ADCs in non-oncologic context, including infections (O’Leary et al., 2023; Tvilum et al., 2023) and auto-immune disorders (Lee et al., 2017; Yasunaga et al., 2017), with promising results showing the possibility of expanding the use of ADCs in various diseases. In this review, we aim to summarize the current knowledge of ADCs and address some key points about their molecular properties, their interaction with tumor mass and TME, their clinical use, toxicities and combinate regimens in cancer treatment. Finally, we will provide an overview on current research on ADCs in other diseases and address the main challenges and limitations in their production and characterization.

## 2 ADCs consist of antibodies linked to cytotoxic payloads via a linker

### 2.1 Antibodies form the scaffold that guides ADCs to target cells

**FIGURE 1:**
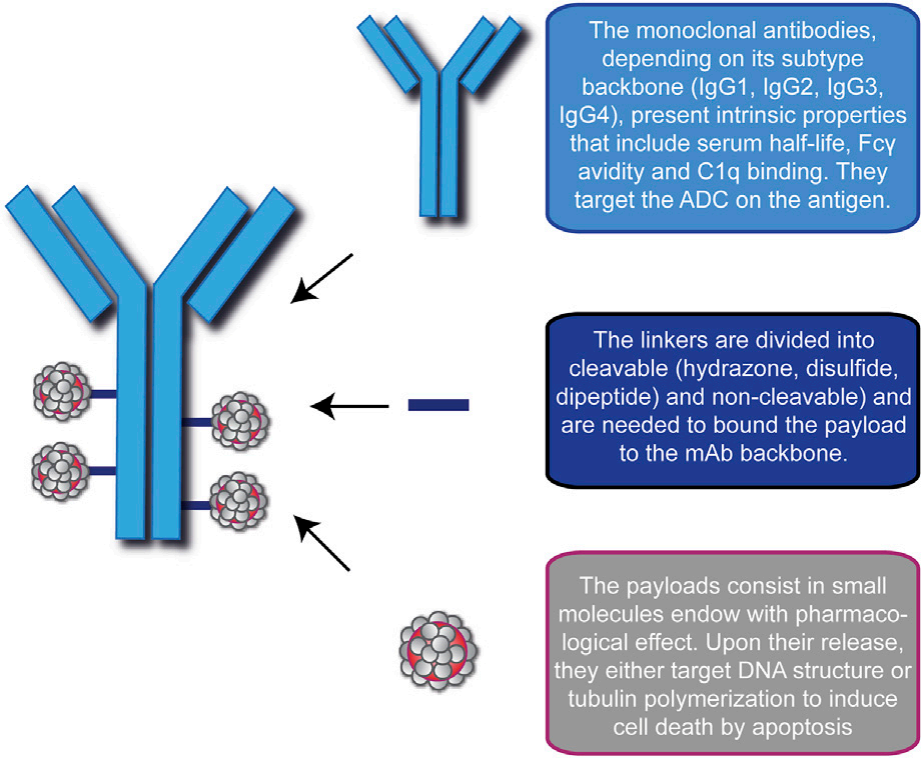
Schematic representation of the modular components of an Antibody-Drug Conjugate (ADC). ADC consists of a monoclonal antibody (blue), a linker (blue lines) and the cytotoxic drugs (gray/red). In this picture, the representative ADC on the left has a Drug-to-Antibody ratio equal to 4. A brief description of the basic function of each module is indicated on the right.

An ADC is a synthetic molecule with pharmacological activity comprising three blocks: a selective mAb, a stable linker, and a potent cytotoxic drug (Figure 1). Together, they ensure tumor-specific targeting and efficient ablation of malignant cells by creating a “new” compound with enhanced therapeutic efficacy.

#### 2.1.1 Full size antibodies are the most used as ADCs scaffolds

An antibody (Ab) or immunoglobulin (Ig) is a Y-shaped glycoprotein produced by plasma cells that presents an intrinsic selective ability to bind to its target (Baah et al., 2021; Pettinato, 2021). Several Abs used in oncology, upon interaction with their target antigens, possess the capacity to influence the biological activity of the tumor mass by modulating survival-related pathways and/or activating potent immune effector functions through three main mechanisms: antibody-dependent cell-mediated cytotoxicity (ADCC), in which the Fc portion of bound Ab is recognized by Natural Killer (NK) cell Fc-receptor and activates the release of lytic factors, complement-dependent cytotoxicity (CDC), in which the interaction between the Fc region and C1q triggers the classic pathway of the complement system leading to cell lysis (Macor and Tedesco, 2007; Macor et al., 2018) and antibody-dependent cellular phagocytosis (ADCP), a mechanism that relies on active macrophages to engulf tumor cells (Peters and Brown, 2015; Chen et al., 2020; Vozella et al., 2021). Similarly, tumor-targeting Abs in ADCs shall be monoclonal and ensure high target specificity and binding affinity, long half-life in plasma, minimal immunogenicity combined with low cross-reactivity, and allow efficient internalization as well as induce direct/indirect killing effects (Peters and Brown, 2015; Dean et al., 2021; Liu and Chen, 2022). According to the amino acid sequence of their heavy chain constant regions, human Igs are classified into 5 isotypes or classes (IgM, IgG, IgA, IgE, and IgD), with IgG being the most abundant in serum. Based on further amino acid variations, this isotype can be subdivided into 4 subtypes (IgG1, IgG2, IgG3, and IgG4). IgG1 consists of the variable heavy (VH) and light (VL) domains in the N-terminal portion of the antibody, the C-domains of the light chains constant region (CL) and the heavy chain constant regions (CH1, CH2, CH3) and the hinge region between the CH1 and CH2 domains (Chiu et al., 2019). To date, its backbone is the most used in ADC preparations because of its serum half-life (∼21 days), high Fcγ receptors avidity, thereby having strong immune activation of Fc-dependent pathways, and more potent complement activation (Yu et al., 2020). Beside these indirect cytotoxic mechanisms, upon the interaction with specific antigens on malignant cells, IgG1-based mAb can also exert direct killing effects by blocking pathways associated to cancer cell proliferation, metastasis and invasiveness (Natsume et al., 2009; Drago et al., 2021). As for the other subclasses, IgG2 plays a role in the response against bacterial capsular polysaccharides but exhibits low Fcγ receptor avidity, low plasma concentration and tends to form covalent dimers that likely lead to aggregation and ADC inefficacy (Zhang et al., 2018). IgG3 protects the body from a range of intracellular bacteria, parasites, and viruses. They are potent mediators of effector functions, including enhanced ADCC and CDC responses, but they are also the subtype with the shortest circulating half-life (Stapleton et al., 2011; Hoffmann et al., 2018), limiting their suitability for ADCs. The IgG4 subtype has a similar half-life to IgG1 and IgG2 but is less efficient at triggering the C1q-related pathway because it has only intermediate affinity for the Fc receptor on phagocytic cells (Spiess et al., 2013; Fu et al., 2022). However, the ability to induce a poor immune response results in a more favorable safety profile compared to the IgG1 backbone, making IgG4 ADCs suitable in cases where antibody-mediated cytotoxicity is not desired (Herbener et al., 2018).

#### 2.1.2 Antibody-fragments represent a new and innovative approach in ADC strategy

A growing number of studies raises questions regarding the usage of full-size IgG moiety in the treatment of solid cancers and points to some critical limitations, particularly on its size (Deonarain et al., 2018; Richards, 2018). As IgG1, which is the most used in ADC synthesis, is quite large (150 kDa) it may hinder the distribution of the drug in the tumor mass and consequently affect in a negative manner ADC pharmacokinetics and the therapeutic outcome. Although this issue may find a partial solution in the tumor-associated leaky vasculature, which shall still allow sufficient pharmacological benefit due to the retention and permeability effect (EPR) (Ferl et al., 2006), its efficiency and the microdistribution of the compound in the tumor depend on many factors, such as ADC preparations and molecular and cellular signatures of the mass (Deonarain et al., 2018; Richards, 2018). Other problems related to scaffold size include systemic accumulation and slowed target-independent clearance rate (Adams et al., 2001; Jain, 2001; Thurber and Dane Wittrup, 2012). Because of these limitations, researchers are seeking new “miniaturized” versions of natural antibodies (also known as antibody-fragments) as a new and smaller drug-conjugatable alternative to expand the ADC therapeutic benefits. These fragments are produced either by proteolytic cleavage of full-size antibodies or by recombinant protein engineering and primarily retain the binding capacity of full-size IgG through the VH and VL regions (Brinkmann and Kontermann, 2017; Kholodenko et al., 2019). They present engineered scaffolds that lack the CH2 domain and Fc region and include three different formulations: the Fab format, a ∼50 kDa structure in which VL and VH are bound to CL and CH1, respectively, and linked by a disulfide bond between the chains, the single-chain variable fragment (scFv), a ∼27 kDa structure in which VH is linked to VL by a short peptide linker, and the diabody, a non-covalent ∼55 kDa dimer scFv consisting of the VH and VL regions linked by a small peptide linker (Xenaki et al., 2017; Deonarain et al., 2018; Kholodenko et al., 2019). In addition, small immunoproteins (SIPs) composed of dimerized scFvs through a CHε4 domain are a fragmentated format developed against fibronectin and other vascular antigens (Perrino et al., 2014). By preserving the targeting capacity of the full-size antibody and combining it with smaller and dynamic formats, antibody-fragments have the potential to overcome some of the major drawbacks of full-size Ig moieties and may represent an innovation in the treatment of solid tumors. To date, promising data showed a remarkable improvement in stability, tumor targeting and penetration and epitope accessibility, particularly in cancers that are still difficult to reach via conventional IgG-based ADCs (Dennis et al., 2007; Thurber et al., 2008). In addition, smaller formats should be better tolerated by the body and produce fewer adverse effects mainly because they do not show cross-reactivity caused by the interaction with Fc receptor and targeted immune cells (Cilliers et al., 2018; Gogesch et al., 2021). If these benefits should be assessed to a greater extent, significant weaknesses can be identified. For example, because of their smaller size and the absence of the Fc domain, without which they cannot trigger the neonatal Fc receptor rescue pathway, antibody-fragments are degraded more rapidly and potentially do not remain in the body long enough to exert sufficient anti-tumor activity. From this perspective, one solution might be to control the dosing regimen by administering higher and/or more frequent doses to achieve a therapeutic effect, but too little is known about the behavior of these formats in the clinic and extensive efforts are underway to improve their feasibility (Deonarain et al., 2018; Deonarain and Xue, 2020). Moreover, the absence of the Fc domain severely limits the possibility of activating the immune system, thus losing an important partner in the fight against the tumor progression. Besides these formulations, scaffolds not based on antibodies are currently being explored (Luo et al., 2022; Kaplon et al., 2023). Nevertheless, though the Ab moiety properties must be carefully considered, the choice of linker and payload are equally important to create the most suitable therapeutic ADC.

### 2.2 Linkers are sequences that connect antibodies to payloads by a chemical bond

In the development of the ADC strategy, linkers represent the technology that ensures a bridge between the mAb and the cytotoxic payload. They are the most modifiable part of an ADC and influence its biophysical and functional properties such as stability, potency, efficacy, and toxicity. The two main purposes of linkers are to prevent the premature release of the cytotoxic drug in the blood circulation and to ensure its efficient release at the target site (Lu et al., 2016; Bargh et al., 2019). Depending on the release mechanisms of the payloads, linkers can be broadly classified into two classes: cleavable and non-cleavable. Recent advances in linker chemistry, including formulations not currently used in clinical ADCs, such as biorthogonal, photo-responsive and Fe(II)-cleavable linkers, are discussed in Su et al., 2021.

#### 2.2.1 Cleavable linkers are versatile and widely employed in ADCs

Cleavable linkers are most commonly used in the synthesis of ADCs and are designed to disengage the cytotoxic drug in response to changes in environmental conditions (pH, redox potential, GSH concentration, or enzymatic activity) that occur between the bloodstream, the tumor cells and the TME niche. They are stable under physiological conditions and, following the internalization of the ADC into the tumor cell, they are rapidly cleaved to ensure selective release of the cytotoxic preparation (Peters and Brown, 2015; Tsuchikama and An, 2018). In addition, these linkers are often cleaved in the TME because of its higher acidity and oxidative stress, making them the most used preparation to affect large solid masses barely impenetrable to full-size antibodies (Ponziani et al., 2020). Cleavable linkers are commonly divided into chemical (hydrazone and disulfide bonds) and enzyme (peptide bonds and glucuronide) cleavage linkers (Bargh et al., 2019). Hydrazone linkers are an example of acid-labile linkers used mainly in hematologic malignancies. They are usually stable in the physiological pH range of the blood circulation and undergo hydrolysis within the acidic microenvironment of the endosomes and lysosomes (pH 4.8–6.2) of the tumor cell (Jain et al., 2015). Similarly, linkers based on disulfide bonds are stable in the bloodstream alkaline environment, but payload release is sensitive to glutathione (GSH), a metabolite whose concentration is much higher in the cytoplasm of cancer cells (Balendiran et al., 2004; Estrela et al., 2006). However, both linkers raised concerns about their non-targeted cytotoxicity (Baah et al., 2021; Fu et al., 2022). Peptide bonds ensure that ADC remains integral in the circulation and enable the release of the cytotoxic drug upon interaction with lysosomal proteases (Tsuchikama and An, 2018), such as cathepsin B, which is generally overexpressed in several tumor cell types (Gondi and Rao, 2013). Peptide linkers are associated with improved serum stability and anticancer activity compared chemical linkers (Lu et al., 2016; Tang et al., 2019; Khongorzul et al., 2020). In addition, linkers based on glucuronide bonds, another type of enzyme-sensitive chemical bridge, are commonly used in ADCs design and rely on cleavage by β-glucuronidase, the level of which is often high in the tumor cellular microenvironment (Kostova et al., 2021).

#### 2.2.2 Non-cleavable linkers avoid non-specific payloads release

Non-cleavable linkers include maleimidocaproyl (MC) and 4-maleimidomethyl cyclohexane-1-carboxylate (MCC) structures and consist of chemical structures that are not fragmented by enzymatic degradation. They are inert to conventional chemicals but allow the release of the cytotoxic drug once the mAb has been completely catabolized by the lysosome. In this way, they release their toxic payload into the tumor target cells without harming normal healthy cells. Due to their chemical synthesis, these linkers offer some advantages over the cleavable alternative, including lower toxicity and longer half-life in plasma (Baah et al., 2021; Fu et al., 2022). On the other hand, their limitations are mainly related to their mechanism of action as they are strongly dependent on an efficient intracellular trafficking and on cellular components with high-expression and internalizing antigens (Lu et al., 2016; Tsuchikama and An, 2018).

### 2.3 Payloads consist of potent cytotoxic agents against tumor cells

As described above, the linker serves as a spacer to connect the mAb to the payload, a cytotoxic drug that must be released in the tumor site to properly exert its pharmacological effects. To be suitable as payloads in ADCs, chemicals shall ideally have low molecular weight and immunogenicity, high stability in the blood circulation and endosomal/lysosomal pathways, and high cytotoxicity (Peters and Brown, 2015; Khongorzul et al., 2020; Baah et al., 2021; Mckertish and Kayser, 2021). Because intravenous administration has shown that only a very small fraction of ADC reaches the tumor (0.1%–2%) (Chari, 2008; Hughes, 2010; Beck et al., 2017), their payloads must be 100- to 1000-fold more effective than the drugs used in chemotherapeutics as free small molecules (Baah et al., 2021). Given that the goal of the ADC strategy is to achieve potent cytotoxic activity, an important attribute to consider in the design process of these compounds is the Drug-to-antibody ratio (DAR), a value that indicates the average number of chemical molecules conjugated to the mAb. For current conjugation methods based on lysine side-chain amidation or mainly on the reduction of cysteine intermediate-chain disulfide bonds, the common DAR values range from 0 (lowest value) to 8 (highest value) (Dan et al., 2018; Wagh et al., 2018; Sun Kang et al., 2021). Nevertheless, *in vivo* experiments have demonstrated that a high DAR value negatively correlates with Ab pharmacokinetics. Although a low DAR implies the loading of poor number of drug molecules and consequently lower therapeutic efficacy, it is worth noting that an average DAR of 2 to 4 results in an ADC with greater anti-cancer activity/efficacy compared to an ADC with higher DAR, likely because the latter is more rapidly cleared from the body when compared to the corresponding average-conjugated counterparts (Hamblett et al., 2004; Strop et al., 2015). Nowadays, cytotoxic payloads usually act as DNA-damaging agents or tubulin inhibitors, but novel potential drugs under investigation include inhibitors of B-cell lymphoma-extra large (Bcl-xL) anti-apoptotic protein, RNA and Niacinamide phosphate ribose transferase (NAMPT) inhibitors, and carmaphycins, inhibitors of proteasome activity (Wang et al., 2023).

#### 2.3.1 DNA-damaging drugs act as crosslinkers, alkylators and topoisomerase inhibitors

The available agents that induce cell death by damaging DNA can act up to picomolar concentrations (Hartley, 2011) and affect both proliferating and non-proliferating cells, so they can potentially contribute to the ablation of the tumor mass by affecting tumor-initiating cells (Cheung-Ong et al., 2013; Bornstein, 2015). From a mechanistic point of view, these chemicals alter the double helix in different ways, e.g., by inducing single/double strand breaks, alkylation and cross-linking of the DNA minor groove, or by inhibiting Topoisomerase I/II and thus replication. Some of them include amanitins (naturally byciclic octapeptides that inhibit RNA Polymerase II action and disrupt RNA and protein synthesis), calicheamicins (DNA-interactive antitumor antibiotics, that cause DNA double-strand breaks and inhibit replication), duocarmycins (natural DNA minor groove alkylating molecules), and pyrrolobenzodiazepines (highly potent DNA minor groove crosslinking agents). Two other compounds that have been used in first-generation ADCs that are worth mentioning are camptothecin (DNA topoisomerase I inhibitor at the replication bubble) and doxorubicin (antibiotic molecule that damages DNA by intercalating into it and generating free radicals) (Sau et al., 2017; Fu and Ho, 2018; Baah et al., 2021; Mckertish and Kayser, 2021).

#### 2.3.2 Tubulin-targeting agents block the mitotic fuse formation and the cell cycle

Tubulin inhibitors block the rapid proliferation of tumor cells at the G2/M cell cycle stage by binding tubulin subunits, leading to cell death by apoptosis. This class includes maytansinoids and auristatins, a family of tubulin-inhibiting cytotoxins that arrest cells in metaphase. The auristatin derivatives monomethyl auristatin E (MMAE) and the less toxic F (MMAF) (Park et al., 2019) are commonly used as payloads in ADC design and exert their function by blocking tubulin polymerization, thereby perturbing microtubule growth and causing cell cycle arrest (Sau et al., 2017; Baah et al., 2021; Mckertish and Kayser, 2021). Looking at the mechanism in detail, microtubule formation involves either nucleation or assembly of the αβ-tubulin heterodimer into a microtubule seed in the cytoplasm (Francisco et al., 2003; Goodson and Jonasson, 2018). As auristatin, by interfering with GTP hydrolysis on the β subunit, causes an excessive and sustained growth of microtubules, they lose the ability to shorten and separate sister chromatids in anaphase, causing cells to freeze in metaphase of mitosis (Waight et al., 2016).

### 2.4 The development of ADC therapeutic strategy goes through three generations of compounds

In the last years the ADC development path brought into the marketplace a dozen ADCs against various hematologic and solid malignancies. Generally, these ADCs have been divided into three generations (first, second, and third) according to the type of mAb, the chemistry of the linker, the mechanism of action as well as the relationship between DAR and the conjugation method (Sau et al., 2017; Fu et al., 2022). Representing the first attempt at a novel therapeutic strategy, the first-generation ADCs caused acute adverse effects, such as hematotoxicity, and morbidity in patients, mainly due to the mAbs backbone, linker chemistry, and target non-specificity rather than the drug itself (Sau et al., 2017). Originally, these biologics utilized mouse-derived and then chimeric mAbs conjugated via unstable linkers to the few weakly potent drugs available in chemotherapy. Unfortunately, after administration, these mAbs were recognized as non-self from the body and inevitably triggered an immune response through the formation of human anti-mouse antibodies (HAMA), often leading to serious immunogenicity problems (Kim and Kim, 2015). In addition, the chemistry of acid-labile linkers, which are quite unstable at bloodstream pH, led to uncontrolled release of payloads (Beck et al., 2017), such as calicheamicin, duocarmycin, and doxorubicin, whose potency was in any case too low to cause cancer cell death. In this context, stochastic conjugation to random lysines did not allow to control DAR and resulted in heterogeneous mAbs mixtures containing unconjugated, partially conjugated, and overconjugated mAbs in unknown proportions, which negatively affected ADC efficacy, limited tumor penetration, and resulted in a narrow therapeutic window (Lucas et al., 2018). Furthermore, antigens were selected even though they lacked tumor-specific expression, resulting in severe systemic off-target effects (Sau et al., 2017; Fu et al., 2022). As expected, based on the limitations of the first-generation ADCs, the second-generation ADCs offered some implementations aimed at improving compound efficacy and largely reducing off-target toxicity. To limit potential side effects associated with the mAb backbone as much as possible, humanized mAbs were preferred over mouse-derived or chimeric mAbs due to the lower immunological response upon administration (Lambert and Chari, 2014). Cleavable linkers have been replaced by the non-cleavable alternative to ensure the stability of ADCs in the blood and to reduce the premature and dangerous release of drugs (Sau et al., 2017; Tsuchikama and An, 2018). In addition, far more potent cytotoxic chemicals have been discovered and selected to induce cell death by attacking DNA structure (disrupting its double helix conformation) and tubulin polymerization (disrupting mitotic fusion formation) (Carter and Senter, 2008; Senter, 2009). Due to the low amount of ADC *in situ*, the IgG1 subtype was preferred over IgG4 because it has better targeting abilities and better conjugation capabilities (Lambert and Chari, 2014). However, despite the introduction of improvements in linker stability and higher drug cytotoxicity, the main weaknesses of this generation of ADCs were still heterogeneous DAR (4–8), rapid clearance for high DAR drugs, off-target toxicity, and drug resistance effects (Sau et al., 2017). What makes the third generation of ADCs the best (so far) is based on fully human mAbs, avoiding the disadvantage of immunogenicity, and optimization in terms of linker stability, payload cytotoxicity, and site-specific conjugation. This new method consists in an evolution of previous ones and was introduced to address heterogeneous DARs and consequently improve ADC pharmacokinetics and utility (Fu et al., 2022). To this end, the synthesis of recombinant Abs bearing engineered cysteine residues enabled the precise bioconjugation of various drugs, resulting in the so-called THIOMAB drug conjugates (TDCs). The THIOMAB antibody technology platform results in a highly stable and effective ADC, whose DAR value is almost uniform (from 2 to 4) and is associated with fewer systemic side effects, improved drug activity, toxicity, and efficacy (Junutula et al., 2008). In addition, despite the potential toxicity due to the high potency of the payloads, these ADCs have lower immunogenicity and hydrophilic linkers, giving patients a more chance to counteract cancer progression (Baah et al., 2021; Fu et al., 2022).

### 2.5 Binding to specific tumor-related targets triggers internalization and cytotoxicity of ADCs

#### 2.5.1 Choosing the right targeting antigen is critical for ADCs killing

Considering that one of the advantages of ADC is that a potent cytotoxic drug can be delivered specifically to cancer cells, the choice of target antigen must be the first consideration in developing this strategy. To take advantage of the maximal therapeutic index of ADCs, the ideal antigen should be a cell surface structure (such as proteins, glycoproteins, or aberrant gangliosides) that is highly or predominantly expressed on tumor cells compared to healthy, normal cells, or at least abundant on malignant, disease-associated cells (Damelin et al., 2015; Peters and Brown, 2015). Ideally, the target proteins should be tumor-specific antigens (TSAs), which are present only on cancer cell types, and/or tumor-associated antigens (TAAs), which are proteins that are highly overexpressed in tumors but rare or sparsely present in normal tissue. In conjunction with this feature, the epitope of the antigen should ideally face the extracellular matrix (rather than the internal site) to ensure easier accessibility and interaction with ADC after diffusing from blood vessels (Tipton et al., 2015; Zhao et al., 2020). In addition, to avoid undesirable systemic side effects and safety issues, the target antigen should be a protein that is anchored to the membrane and not secreted into the blood circulation. If this were the case, ADC would promote unwanted binding outside the tumor and thus reduce the anticancer effect on malignant cells (Ritchie et al., 2013; Zhao et al., 2020). Nevertheless, after the ADC interaction, the optimal antigen should ensure proper internalization of the antigen- ADC complex into the endosomal/lysosomal pathway, leading to drug release and ultimately cytotoxicity. It should be noted that the speed and efficiency of the internalization process strictly depend on the nature of the target, the type of the epitope, and the payload conjugated to ADC (Carter and Senter, 2008; Donaghy, 2016; Fu et al., 2022). Finally, since the tumor mass and its surrounding microenvironment (TME) are tightly coupled and constantly communicate with each other, TME components have been targeted as novel potential ADC targets (see below in the text) (Andersen, 2023).

#### 2.5.2 The mechanism of action of ADCs requires internalization to exert antitumor cytotoxicity

**FIGURE 2:**
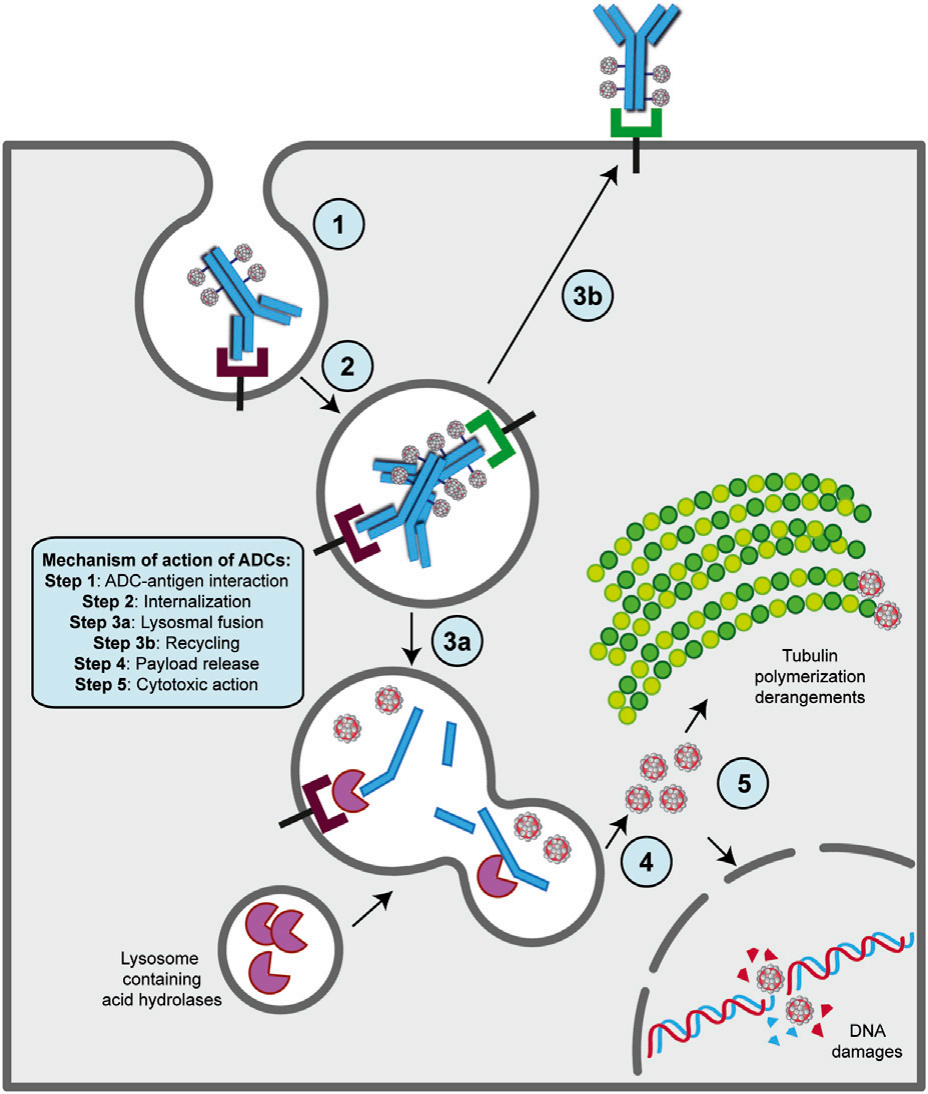
Mechanism of action of ADC strategy. The major steps of the process are indicated on the figure. Basically, following ADC-target interaction on the surface of cancer cell (step 1), this complex undergoes receptor-mediated endocytosis and enters the endosomal/lysosomal pathway until the payload is released in the cytoplasm (steps 2, 3a and 4). Then, the drug can exert its killing activity either damaging DNA structure in the nucles or derange mitotic fuse polymerization (step 5), leading to cell death by apoptosis. A fraction of ADCs binds to FcRn receptors in the early step on endosomal/lysosomal pathway and get transported out of the cell (step 2a and 3b).

The presence of a mAb targeting an antigen that is either specifically present on cancer cell types or highly overexpressed during carcinogenesis is a fundamental requirement for the ADC strategy (Alley et al., 2010; Damelin et al., 2015). With this in mind, the mechanism of action of ADCs is quite simple and allows for therapeutic action against the disease as well as potent on-target cytotoxicity (Figure 2) (Peters and Brown, 2015; Khongorzul et al., 2020; Drago et al., 2021; Samantasinghar et al., 2023). After administration, usually by intravenous injection to preserve drug functionality (Nolting, 2013), the mAb portion of the ADC binds to the target antigen on cancer cells, and after internalization of the antigen- ADC complex by receptor-mediated endocytosis, the newly formed early endosome matures into a late endosome that ultimately fuses with the lysosome. In this cellular compartment, acidic or redox conditions combined with the presence of proteases (cathepsin B, plasmin, etc.) allow the detachment of the cytotoxic payload from its mAb carrier, whereupon the drug diffuses into the cell and leads to cell death by attacking DNA structure or microtubule polymerization (Peters and Brown, 2015; Khongorzul et al., 2020; Drago et al., 2021; Samantasinghar et al., 2023). Of note, the IgG subtype that is widely employed in ADC synthesis can be rescued from the endosomal/lysosomal degradation pathway and recycled outside cells through interaction with the neonatal Fc receptor (FcRn), an IgG receptor involved in the regulation of IgG turnover. Therefore, this FcRn-mediated transcytosis into the extracellular space, although involving only a small percentage of the internalized ADC -complex, can potentially enhance the clearance of ADC and thus reduce its therapeutic index (Xu, 2015). On the other hand, if the small molecule is permeable to the cell membrane, it can partially diffuse back into the extracellular matrix and enter neighboring cells regardless of the expression level of the antigen, causing a “bystander effect” (Staudacher and Brown, 2017). This phenomenon, by altering components of the TME, such as neovascular endothelial cells and/or cancer-associated fibroblasts (CAFs), could further enhance the killing effect of ADC, especially in cancer lesions with high heterogeneous target expression (Gerber et al., 2009; Purcell et al., 2018; Szot et al., 2018). Moreover, the therapeutic strategy of ADCs involves other killing mechanisms to ensure efficacy against cancer. In several contexts, it has been shown that the interaction of the mAb with its specific target can directly cause potent inhibition of downstream signaling pathways triggered by antigen receptor stimulation (Albanell et al., 2003; Vu and Claret, 2012; Marei et al., 2022). While the Fab fragment of the carrier is bound to the target epitope on the malignant cell, the Fc portion of the same mAb can interact with the FcR on NK cells and macrophages, triggering ADCC and ADCP, respectively, as well as the C1q component of the complement system, triggering CDC (Junttila et al., 2011; Tai et al., 2014; Redman et al., 2015).

### 2.6 The TME offers new potential targets to ADCs strategy

Most of the ADCs in preclinical and clinical development target TAAs or TSAs localized mainly on the cancer mass (Sau et al., 2017; Tong et al., 2021). Compared to hematologic malignancies, solid tumors thrive in a complex and dynamic entity called the TME, whose composition can vary widely depending on the tumor type. Key features of the TME generally include abundant extracellular matrix, stromal cells (e.g., cancer-associated fibroblasts, CAFs), new and abnormal blood vessels, and immune cells, the latter capable of infiltrating the tumor mass and exerting pro- and anti-tumor functions (Anderson and Simon, 2020; Baghban et al., 2020). Particularly in the early stages of tumor growth, a bidirectional and complex interaction exists between cancer cells and the TME components through the release of soluble factors that promote the survival of the tumor mass, its local invasion and subsequent metastatic spread (Visser and Joyce, 2023). In this sense, the TME supports the basic needs of the tumor through a neo-angiogenesis program that removes metabolic waste and, most importantly, restores oxygen and nutrient supply to the mass (Anderson and Simon, 2020; Visser and Joyce, 2023; Wang et al., 2023). Given the close relationship between these two entities, TME-associated antigens (TMAs), i.e., proteins that are dysregulated on non-malignant cells within the TME, offer new potential targets for the treatment of solid tumors as they differentiate from more traditional tumor antigens (Andersen, 2023). Major TMAs include chemokines and cytokines, transcription factors, metabolic enzymes, and checkpoint molecules. Some of their advantages lie in their overexpression on endothelial, stromal, and immune cells, whereas they are rare or very low in healthy tissues, and in their easier accessibility to ADCs when administered into the bloodstream, especially to antigens present on neo-vessels or stromal cells. Furthermore, because TME components are distinct from cancer cells, they are less susceptible to resistance mechanisms caused by inefficient DNA repair mechanisms (Agrafiotis et al., 2022; Ceci et al., 2022; Andersen, 2023). The development of agents against these antigens not only weakens the tumor mass but also provides the opportunity to modulate the TME itself, making it less immunotolerant and more susceptible to tumor ablation (Andersen, 2023). Preclinical and clinical evidence suggest that cell types/factors belonging to the tumor extracellular matrix and neo-blood vessels may be valuable choices for new ADC target antigens (Peters and Brown, 2015; Xiao and Yu, 2021). For instance, an ADC targeting stromal cells may cause cell death by altering the concentration of growth factors in the tumor niche or induce hypoxia and nutrient deprivation by binding to an antigen on the neo-vasculature (Jain, 2005; Mahadevan and Von Hoff, 2007; Xiao and Yu, 2021). In this regard, it is worth mentioning the effect of the “binding site barrier” (BSB), a phenomenon that occurs between mAb and cell populations near blood vessels and retains part of the ADC near them, reducing the penetration of Ab into the tumor mass (van Dongen, 2021). However, most TMAs have been identified on cells of the immune system and targeting them offers an innovative anti-cancer therapeutic approach achieved by promoting effector cell proliferation, the anti-cancer cytokine/chemokine production, and overall survival to create a new immune-hostile tumor niche with reduced neo angiogenesis (Gajewski et al., 2006; Labani-Motlagh et al., 2020; Andersen, 2023; Del Prete et al., 2023). To date, some TMAs targeted by novel ADCs include CD74, an MHC class chaperone II targeted by the ADC STRO-001, currently in phase I in the treatment of B-cell malignancies (NCT03424603) (Le et al., 2023) and CCR7, a chemokine receptor targeted by the novel ADC JBH492 in non-Hodgkin lymhoma and chronic lymphocytic leukemia patients (NCT04240704). In addition, Camidanlumab tesirine, also known as ADCT-301, is an ADC in phase 1/2 for the treatment of classical Hodgkin’s lymphoma (cHL) and non-HL (NCT02432235) (Hamadani et al., 2021), and CD276, an immune checkpoint overexpressed during pathologic angiogenesis and an interesting candidate target of different ADCs in advanced solid tumors (NCT04145622, NCT03729596 and NCT03595059) (Ceci et al., 2022; Ziogas et al., 2023).

### 2.7 Cancer creates different ways to escape the effectiveness of ADCs

A well-known feature of cancers is their ability to overcome the efficacy of therapeutic approaches, making them susceptible to various mechanisms of resistance. The evasion mechanisms developed by malignant cells can be divided into down-/high-regulation of antigen, the presence of drug efflux pumps, defects in lysosomal functions, and deregulation of signaling pathways involved in cell cycle progression and apoptotic dysregulation (Shefet-Carasso and Benhar, 2015; Loganzo et al., 2016; García-Alonso et al., 2018). In this context, an association between antigen levels and the efficacy of ADC treatment with brentuximab vedotin was observed, whose multiple treatment cycles correlated with CD30 downregulation and consequently stronger tumor resistance to MMAE (Chen et al., 2015). In another study, the cancer cell line JIMT1-TM showed long-term resistance to the drug after repeated administration of anti-human epidermal growth factor receptor 2 (anti-HER2) transtuzumab maytansinoid, as the level of HER2 protein decreased (Loganzo et al., 2015). Nevertheless, upregulation of CD33 antigen in the blood limited gemtuzumab ozogamicin penetration into the bone marrow, suggesting that elevated CD33 levels still negatively affect treatment and likely reduce drug exposure (van der Velden et al., 2004). Another non-negligible mechanism of resistance relies on a family of transmembrane proteins called ABC transporters (Zheng et al., 2021). These transmembrane proteins act as drug efflux pumps, causing various chemicals, including those used as payloads, to be excreted from the cancer cell, making it resistant or at least less susceptible to treatment (Zheng et al., 2021). This mechanism has been observed in AML cells, in which overexpression of the ABC-family member MDR1 made them resistant to gemtuzumab ozogamicin (Matsumoto et al., 2012; Senter and Sievers, 2012) and in breast carcinoma cells, in which cyclic dosing of TDM-1 induced an increase in ABCC1 transporter levels (Loganzo et al., 2015). Another escape mechanism involves lysosomal acidification. After administration of ADC and internalization, linkers are cleaved by lysosomal acid hydrolases to subsequently release the cytotoxic agent into the cytoplasm of cancer cells. However, upon persistent treatments malignant cells may acquire the ability to disrupt this process by altering the pH of the lysosomal compartment to slow the catabolic activity of their proteases, a process that has been demonstrated in HER2-positive breast cancer clones resistant to long-term T-DM1 (Ríos-Luci et al., 2017; García-Alonso et al., 2018). Nevertheless, perturbations in signaling pathways involved in cell cycle regulation and alterations in apoptotic regulation may also modulate tumor cell sensitivity to ADC (Collins et al., 2019). In T-DM1-resistant HER2-positive breast cancer cells, an increase in cyclin B levels, a protein required for the G2/M cell cycle transition, has been observed. This upregulation at the protein level could affect cell cycle dynamics by altering sensitivity to treatment with ADC (Sabbaghi et al., 2017). Moreover, in AML cells, activation of a related pathway (PI3K/Akt) was associated with lower efficacy of gemtuzumab ozogamicin and a deletion in the PTEN pathway was associated with trastuzumab low effectiveness (García-Alonso et al., 2018). Of note, in the same hematologic tumor, overexpression of members of the anti-apoptotic BCL-2 and BCL-X families plays a role in sensitivity to gemtuzumab ozogamicin (Godwin et al., 2020).

### 2.8 Clinically approved ADCs for the treatment of hematologic and solid malignancies

The ADC strategy represents a highly successful therapeutic alternative in cancer treatment. The excellent ability to deliver a pharmacological compound *in situ* by drawing the mAb of choice along with various linkers and chemical alternatives represented an innovative development compared to mAb-only based therapy and traditional chemotherapy. As mentioned earlier, although the development of ADC remains challenging in terms of drug safety, efficacy, and targeting, the development of new and more precise technologies, as well as the identification of new targets and components, has led to an explosion in the use of ADC in clinical oncology (Drago et al., 2021; Dumontet et al., 2023; Fuentes-Antrás et al., 2023). To date, hundreds of ADCs are in clinical trials, and 15 of them have been approved by the FDA, the European Medicines Agency (EMA), and/or other government agencies and launched into the marketplace for the treatment of hematologic malignancies and solid tumors. Over the past 23 years, the following ADCs have been developed for the treatment of hematologic tumors: Gemtuzumab ozogamicin (Mylotarg®), Brentuximab vedotin (Adcetris®), Inotuzumab ozogamicin (Besponsa®), Polatuzumab vedotin (Polivy®), Belantamab mafodotin (Blenrep®), Loncastuximab tesirine (Zynlonta®) and Moxetumomab pasudotox (Lumoxiti®), an ADC, which uses an immunotoxin rather than a chemotherapeutic agent as a payload. The ADCs currently approved for solid tumor therapy are Ado-trastuzumab emtansine (Kadcyla®), Fam-trastuzumab deruxtecan (Enhertu®), Enfortumab vedotin (Padcev®), Sacituzumab govitecan (Trodelvy®), Tisotumab vedotin-tftv (Tivdak®), Mirvetuximab soravtansine (ELAHERE®), Disitamab vedotin (Aidixi®), and Cetuximab sarotalocan (Akalux®) (Fu et al., 2022; Kaplon et al., 2023; Yao et al., 2023). A brief description of each agent is provided below and key characteristics are listed in Table 1. In addition, Supplementary Table 1 provides the recruiting clinical trials on the use of novel ADCs being investigated for the treatment of cancer.

**TABLE 1:** FDA/EMA approved ADCs in clinical oncology. e early; m metastatic; AML acute myeloid leukemia; HL Hodgkin lymphoma; sALCL systemic Anaplastic Large Cell Lymphoma; B-cell prec. L B-cell precursor leukemia; DLBCL diffuse large B-cell lymphoma; MM multiple myeloma; HCL hairy cell leukemia; BC breast cancer; UC urothelial cancer; NSCLC non-small-cell lung cancer; GC gastric cancer; GOJ gastro-oesophageal junction cancer; TNBC triple negative breast cancer; CC cervical cancer; OC ovarian cancer; HNSCC Head and neck squamous cell carcinoma. The detailed description of the treatment for each disease is described in the text. *: withdraw from market in 2022. **: Aidixi® and Akalux® have not received FDA/EMA approval yet but Akalux® is approved by PMDA (Pharmaceuticals and Medical Devices Agency of Japan) and Aidixi® by NMPA (National Medical Products Administration of China).

ADC	Manufacturer	Trade name®	Target	FDA/EMA approval	Cancer
Gemtuzumab ozogamicin	Pfizer	Mylotarg	CD33	2000(2017)/2018	AML
Brentuximab vedotin	Seagen, Takeda Pharma	Adcetris	CD30	2011/2012	HL; sALCL
Inotuzumab ozogamicin	Pfizer	Besponsa	CD22	2017	B-cell prec. ALL
Polatuzumab vedotin	Genentech	Polivy	CD79b	2019/2020	DLBCL
Belantamab mafodotin	GlaxoSmithKline	Blenrep	BCMA	2020*	MM
Loncastuximab tesirine	ADC Therapeutics	Zynlonta	CD19	2021/2022	large B-cell prec. L
Moxetumomab pasudotox	AstraZeneca	Lumoxiti	PE38	2018	HCL
Ado-Trastuzumab Emtansine	Genentech	Kadcyla	HER2	2013	HER2+ e/m BC
Enfortumab vedotin	Astellas Pharma US, Seagen	Padcev	NECTIN4	2019/2022	mUC
Fam-trastuzumab Deruxtecan	Daichii Sankyo	Enhertu	HER2	2019/2021	HER2+ BC; NSCLC; GC/GOJ
Sacituzumab govitecan	Gilead Sciences	Trodelvy	TROP2	2020/2021	TNBC; mUC
Tisotumab vedotin-tftv	Seagen	Tivdak	TF	2021 (only FDA)	CC
Mirvetuximab Soravtansine	ImmunoGen	ELAHERE	FRα	2022 (only FDA)	OC
Disitamab Vedotin	Remegen	Aidixi**	HER2	**—**	UC; GC
Cetuximab Sarotalocan	Rakuten Medical	Akalux**	EGFR	**—**	HNSCC

#### 2.8.1 Hematologic malignancies

##### 2.8.1.1 Gemtuzumab ozogamicin

Gemtuzumab ozogamicin (Mylotarg®; Pfizer) was the first ADC ever developed and clinically approved by the FDA in 2000 and by the EMA in 2018. As a first-generation ADC, it was based on a humanized anti-CD33 IgG4 antibody linked to the DNA-interactive agent calicheamicin (or ozogamicin) via a hydrazone-cleavable linker bound to surface lysines (average DAR 2–3). It was indicated for the treatment of relapsed/refractory acute myeloid leukemia (AML) though it was withdrawn in 2010, but reapproved at a lower dose in 2017, because patients suffered severe toxicity problems likely due to the higher dose (Fu et al., 2022). Following administration, Mylotarg binds to the CD33 transmembrane glycoprotein on AML cells, and upon internalization, a precursor of calicheamicin is released through hydrolysis of its linker. The active form of the drug then exerts a cytotoxic activity by binding to DNA and breaking its conformation to cause cell cycle arrest and cell death. Of note, the hydrophobic nature of calicheamicin enables a bystander killing of cells in the TME that are negative for the CD33 target antigen (Coats et al., 2019; Kayser and Levis, 2022).

##### 2.8.1.2 Brentuximab vedotin

Brentuximab vedotin (Adcetris®; Seagen, Takeda Pharma) was approved by the FDA in 2011 and by the EMA in 2012 as monotherapy for the treatment of systemic anaplastic large cell lymphoma (sALCL) and in 2018 in combination with chemotherapy for relapsed/refractory Hodgkin lymphoma (HL). It consists of a chimeric IgG1 targeting CD30, a cell membrane protein of the tumor necrosis factor receptor family, on cancer cells and is cysteine conjugated to MMAE (DAR equals 4) through a protease-cleavable linker (Mckertish and Kayser, 2021). After interacting with its target, Adcetris enters the endosomal/lysosomal pathway via clathrin-dependent endocytosis, where its linker is cleaved by acid hydrolases to release MMAE in the cytoplasm. The drug interferes with microtubule polymerization and induces apoptosis and cell death. Like calicheamicin, MMAE exerts its cytotoxic effect on neighboring CD30-negative cells using the bystander killing effect, suggesting that the efficacy of Adcetris in heterogeneous lymphomas *in vivo* may be related to this effect (Katz et al., 2011; Scott, 2017).

##### 2.8.1.3 Inotuzumab ozogamicin

Inotuzumab ozogamicin (Besponsa®; Pfizer) was approved by the FDA and EMA in 2017. It targets CD22, an antigen expressed on relapsed/refractory B-cell precursors in acute lymphoblastic leukemia (ALL). It consists of a humanized IgG4 mAb linked to calicheamicin via an acid-cleavable linker attached to lysine residues. It has a DAR, ranging from 5 to 7. From a mechanistic perspective, it acts similarly to gemtuzumab ozogamicin in that it is based on the same Ab backbone and loaded with the same drug (Dahl et al., 2016; Garrett et al., 2019; Lanza et al., 2020).

##### 2.8.1.4 Polatuzumab vedotin

Polatuzumab vedotin (Polivy®; Genentech) contains a humanized IgG1 anti-CD79b, a component of the B-cell receptor conjugated to MMAE via the same organic bridge used in the synthesis of brentuximab vedotin. The conjugation method was via engineered cysteines utilizing the THIOMAB system and has a DAR of 3.5. The clinical use of Polivy has been approved by the FDA in 2019 and by the EMA in 2020 for the treatment of adult patients with relapsed/refractory diffuse large B-cell lymphoma (DLBCL) in combination with bendamustine and rituximab (anti-CD20 mAb) (Sehn et al., 2022; Varma et al., 2022). Upon administration, this agent is internalized into cancer cells and proteolytically cleaved to release MMAE, which causes apoptotic cell death by inhibiting tubulin polymerization (Deeks, 2019; Kaplon et al., 2020).

##### 2.8.1.5 Belantamab mafodotin

Belantamab mafodotin (Blenrep®; GlaxoSmithKline) was approved by the FDA and EMA for the treatment of refractory/relapsed multiple myeloma in 2020 (MM) but was withdrawn in 2022 because it did not meet FDA standards (https://www.myeloma.org/news-events/withdrawal-blenrep-us-market). The mAb portion of this ADC is unique in that it consists of a humanized Fc-afucosylated IgG1, a modification that enhances binding and cytotoxicity of the ADC. From the mAb backbone, a cysteine-bound, non-cleavable linker bridges the mAb with the cytotoxic payload MMAF. Its DAR is 4. The target of Blenrep is B cell maturation antigen (BCMA), a member of the tumor necrosis factor receptor family that is overexpressed in mature B lymphocytes and plasma cells (Chen et al., 2020; Yu et al., 2020). As with other ADCs, BCMA targeting internalizes ADC and degrades mAb to release MMAF, a tubulin inhibitor, into the cytoplasm, where it blocks cancer cell cycle progression and leads them to death through apoptosis (Seckinger et al., 2017; Kaplon et al., 2020).

##### 2.8.1.6 Loncastuximab tesirine

Loncastuximab tesirine (Zynlonta®; ADC Therapeutics) targets CD19 and received accelerated approval from the FDA in 2021 and from the EMA in 2022 for relapsed/refractory B-cell lymphoma after two or more lines of systemic therapy, including DLBCL not otherwise specified, DLBCL from low-grade lymphoma, and high-grade B-cell lymphoma. Zynlonta targets CD19, a transmembrane protein commonly expressed in all B cell lineages, and consists of a humanized IgG1 mAb linked to SG3199 (a dimeric PBD alkylating agent) via an enzymatically cleavable linker (DAR of 2–3) (Lee, 2021). The payload exerts its pharmacological benefit by irreversibly binding to DNA and generating a strong adduct that inhibits DNA synthesis and causes cell death. Because SG3199 exhibits cytotoxicity in the picomolar range, it is the most toxic drug currently available on the market ADC. Currently, Zynlonta is the only anti-CD19 drug approved ADC for relapsed/refractory DLBCL as a single agent (Zammarchi et al., 2018; Fu et al., 2022).

##### 2.8.1.7 Moxetumomab pasudotox

Moxetumomab pasudotox (Lumoxiti®; AstraZeneca) is not widely considered an ADC because its payload consists of a fragment of *Pseudomonas aeruginosa* exotoxin A called PE38. However, since it uses the same targeting mechanism based on mAb, we would still like to consider it as part of the ADC biocompound family. It was approved by the FDA in 2018 for patients with refractory/relapsed hairy cell leukemia (HCL) who have not received at least two systemic therapies. It was granted marketing authorization in the EU in 2021. Lumoxiti is based on an anti-CD22 mouse IgG1 mAb carrying a cleavable linker bound to the immunotoxin PE38. After interaction, internalization, and cleavage, PE38 is released into the cell cytoplasm and acts by blocking translation and inducing cell apoptosis (Kreitman and Pastan, 2011; Kreitman, 2019; Kang, 2021).

#### 2.8.2 Solid tumors

##### 2.8.2.1 Trastuzumab-based ADCs

Since FDA approval of rituximab for the treatment of non-HL in 1997 (Leget and Czuczman, 1998), several mAbs have been investigated and achieved approval in clinical oncology. Considering the mAbs that form the scaffold of approved ADCs, trastuzumab is an example of an anti-cancer molecule used in combination therapy or alone, and also represents the carrier motif in two preparations ADC. Trastuzumab (Herceptin®) is a humanized IgG1mAb that binds to the extracellular domain of HER2, a tyrosine kinase receptor that is upregulated in 20% of breast cancer (BC) patients, preventing its homodimerization and thereby blocking its intracellular signaling (Greenblatt and Khaddour, 2023). Because of its role in cell growth, survival, and differentiation, HER2+ breast cancers tend to grow and spread more aggressively than HER2 negative tumors (Iqbal and Iqbal, 2014). Trastuzumab was approved by the FDA in 1998 for the treatment of HER2+ BC. It improved overall survival (OS) and progression-free survival (PFS) (Albanell and Baselga, 1999; Goldenberg, 1999), but its administration was also associated with risk of cardiac toxicities, such as left ventricular ejection fraction (LVEF) decline and congestive heart failure (Bouwer et al., 2020). In the U.S. it is approved for HER2+ BC in adjuvant therapy (with anthracyclines and taxane) and for metastatic HER2+ BC in monotherapy or in combination with chemotherapeutics, tyrosine kinase inhibitors (TKIs), and immunotherapy. It is also used in a combination regimen for HER2+ gastric cancer (Dumontet et al., 2023). Trastuzumab is administered by intravenous infusion, and the dosing regimen can be adjusted depending on the stage of tumor growth (Greenblatt and Khaddour, 2023). Nowadays, the trastuzumab backbone has been used for the synthesis of two FDA- and EMA-approved ADCs, ado-trastuzumab emtansine (T-DM1, Kadcyla®; Genentech) and fam-trastuzumab deruxtecan (T-Dxd, Enhertu®, Daiichi Sankyo, Astrazeneca), which have improved the OS in the second and third-line settings and are currently used for the treatment of HER2+ early/metastatic and HER2+ low BC, respectively (Ferraro et al., 2021; Rassy et al., 2022).

##### 2.8.2.1.1 Ado-trastuzumab emtansine

Ado-trastuzumab emtansine (Kadcyla®; Genentech) is based on a humanized IgG1 linked to emtansine via a non-cleavable linker attached to the lysine residues. Its average DAR is 3.5 (Mckertish and Kayser, 2021). After interaction with HER2 antigen, Kadcyla is internalized by endocytosis and reaches the lysosome, where IgG1 is completely proteolytically degraded. Subsequently, lysine-MCC-DM1, a DM1-containing metabolite, is released into the cytosol, where it disrupts the microtubule network and causes cell death. Interestingly, lysine-MCC-DM1 has similar toxicity to DM1 but cannot exert its pharmacological effect via the bystander killing effect due to its charge at neutral pH (Barok et al., 2014; Lambert and Chari, 2014). T-DM1 received FDA approval in 2013 for the treatment of advanced HER2+ BC based on data from the EMILIA clinical trial (Phase III). This study evaluated the efficacy of T-DM1 compared to capecitabine and lapatinib in patients with HER2+ BC previously treated with transtuzumab and taxane chemotherapy. Results based on 911 included patients were favorable for T-DM1, whose administration resulted in an improvement in objective response rate (ORR) (43.6% vs. 30.8%), median PFS (9.6 months vs. 6.4 months; *p* < 0.001), and median OS (29.9 vs. 25.9 months, *p* < 0.001) after a median follow-up of 47.8 months (Blackwell et al., 2012; Diéras et al., 2017). A few years later, positive results from the KATHERINE clinical trial (phase III) established the novel use of T-DM1 as adjuvant therapy for patients with early-stage HER2+ BC with residual disease after neoadjuvant treatment (taxane and trastuzumab). Among the 1,486 patients who met criteria, those who received T-DM1 showed a significant 50% improvement in invasive disease-free survival (IDFS) at 3 years compared with patients treated with trastuzumab alone in the control arm (88.3% vs. 77%, *p* < 0.001) (von Minckwitz et al., 2019; Wedam et al., 2020; Mamounas et al., 2021). From the data collected in these two studies, T-DM1 exhibits stronger therapeutic efficacy compared to chemotherapeutic agents and trastuzumab alone in HER2+ early or metastatic BC, likely because this ADC preserves the antineoplastic functions of trastuzumab and adds a novel cytotoxic effect (Ferraro et al., 2021). Remarkably, the superior benefit of T-DM1 was associated with manageable side effects, mostly grade 1 or 2, as only a small percentage of included patients reported elevations in liver enzymes aspartate transaminase (AST) and alanine transaminase (ALT) and thrombocytopenia (Diéras et al., 2017).

##### 2.8.2.1.2 Fam-trastuzumab deruxtecan

Fam-trastuzumab Deruxtecan (Enhertu®; Daiichi Sankyo) is the second ADC HER2-targeting drug approved by the FDA. T-Dxd was approved by the FDA in 2019 and by the EMA in 2021 for the treatment of unresectable or metastatic HER2+ breast cancer (after patients have received two or more anti-HER2 therapies), non-small cell lung cancer, and for locally advanced or metastatic HER2+ gastric or gastroesophageal junction adenocarcinoma, after a trastuzumab-based therapy. It consists of a humanized IgG1-mAb carrying Dxd, a more potent DNA topoisomerase I inhibitor than SN-38, as a cytotoxic payload via an enzymatically cleavable linker. It has a DAR of 7 or 8 (Mckertish and Kayser, 2021; Fu et al., 2022; Dumontet et al., 2023). After internalization of the HER2-Enhertu complex and cleavage, Dxd blocks DNA topoisomerase I, an enzyme that controls and alters the topological state of DNA during transcription, leading to cell death. Compared to Kadcyla, which has the same target, Enhertu has several improvements related to the novel cysteine-conjugated peptide linker, higher DAR and cytotoxicity of the drug, which is more potent and hydrophobic to increase the bystander killing effect on neighboring cells (Kaplon et al., 2020; Shitara et al., 2021). This is essential for extending cytotoxic activity to cells with low or heterogeneous HER2 levels. In this sense, the bystander-killing effect achieved by T-Dxd allows for a better therapeutic response and thus greater cytotoxicity in tumors refractory to its T-DM1 counterpart (Ferraro et al., 2021). In 2019, T-Dxd received accelerated FDA approval based on positive results from the single-arm DESTINY -Breast01 trial (Phase II). In this study, a cohort of 184 female patients with HER2+ metastatic BC who had received two or more prior lines of therapy, including T-DM1, was enrolled to test the efficacy of T-Dxd. Interestingly, 60.9% of patients showed an objective response, and the median duration and median response were 16.4 months and 14.8 months, respectively, with a median time response of 1.6 months (95% CI) (Modi et al., 2020; 2021). In addition, the efficacy of T-Dxd and T-DM1 was evaluated in the DESTINY -Breast 03 trial (phase III), which enrolled 524 patients with HER2+ metastatic BC previously treated with trastuzumab and taxane. Randomization data showed superior efficacy of T-Dxd in terms of ORR (79.9% vs. 34.2%), PFS (not reached vs. 6.8 months, *p* < 0.001), and OS (94.1% vs. 85.9%) (Cortés et al., 2021; 2022). In general, hematotoxicity, nausea, and fatigue were the most common grade ≥3 adverse effects observed in patients treated with T-Dxd. Of note, treatment with T-Dxd was also associated with pulmonary toxicity and, in particular, interstitial lung disease (ILD), a group of respiratory diseases that require careful management and may lead to treatment discontinuation (Diéras et al., 2017; Cardoso et al., 2020). Finally, the DESTINY-Breast04 trial evaluated the use of T-Dxd *versus* physician’s choice chemotherapy in 577 patients with low HER2+ metastatic BC who had received prior chemotherapy in the metastatic setting or developed a recurrence within 6 months of completing adjuvant chemotherapy. The results of this study showed that administration of T-Dxd resulted in significantly longer median progression-free (9.9 months *versus* 5.1 months) and overall survival (23.4 months *versus* 16.8 months) than pharmacologic therapy in enrolled patients (Modi et al., 2022).

##### 2.8.2.2 Enfortumab vedotin

Enfortumab vedotin (Padcev®; Astellas Pharma US, Seagen) is a fully human IgG1 conjugated to the microtubule inhibitor MMAE via a protease-cleavable linker on cysteine residues and has a DAR of 3.8 (Joubert et al., 2020). It targets nectin-4, a transmembrane protein involved in multiple cellular signaling pathways, including cell adhesion, proliferation, and migration, and is overexpressed in several malignancies. in 2019, Padcev was approved by the FDA for locally advanced or metastatic urothelial carcinoma following Pt-containing therapy and a PD -1 or PD -L1 inhibitor (Alt et al., 2020; Chang et al., 2021) and received EU-wide marketing approval in 2022.

##### 2.8.2.3 Sacituzumab govitecan

Sacituzumab govitecan (Trodelvy®; Gilead Sciences) is an ADC consisting of a humanized IgG1 mAb targeting Tumor-associated calcium signal transducer 2 (TROP2), a transmembrane glycoprotein involved in cell self-renewal, proliferation, invasion, and survival, and plays an important role in intracellular calcium signaling. It is generally overexpressed in most solid tumors, including triple negative breast cancer (TNBC) (Furlanetto et al., 2022). The mAb harnesses to malignant cells SN-38, a DNA topoisomerase I inhibitor that causes DNA breaks and ultimately cell death. IgG1 and payload are connected via an acid-cleavable linker bound to cysteine residues with a DAR between 7 and 8 (Tong et al., 2021). Trodelvy was approved by the FDA in 2020 and by the EMA in 2021 for the treatment of locally advanced or metastatic TNBC in patients who have received at least two prior therapies and in locally advanced or metastatic urothelial carcinoma following Pt-containing therapy and a PD -1 or PD -L1 inhibitor (Mehanna et al., 2019; Bardia et al., 2021; Tong et al., 2021).

##### 2.8.2.4 Tisotumab vedotin-tftv

Tisotumab vedotin-tftv (Tivdak®; Seagen) consists of a fully human IgG1, an enzymatically cleavable linker, MMAE as a payload, and a DAR equal to 4. It targets tissue factor (TF), a membrane protein related to cancer metastasis and invasiveness that is highly expressed in various solid tumors. Being the last ADC on the market, it was approved by the FDA in 2021 for the treatment of relapsed/refractory metastatic cervical cancer with disease progression during or after chemotherapy (Liu et al., 2011; Alley et al., 2019; Heitz et al., 2023).

##### 2.8.2.5 Mirvetuximab soravtansine

Mirvetuximab soravtansine (ELAHERE®; ImmunoGen) was approved by the FDA in 2022 for the treatment of adult patients with Folate Receptor α (FRα)-positive, platinum-resistant epithelial ovarian cancer who have previously received 1–3 systemic therapies. It targets FRα, a member of the folate receptor family that is overexpressed on several epithelial-derived cancer cells (Macor et al., 2006). It consists of a chimeric mAb bound via a cleavable linker to DM4, a potent tubulin targeting agent that belongs to the maytansinoids. Like other ADC, the drug exerts its cytotoxic effect after internalization into cancer cells, leading to their death by blocking their mitotic fuse formation (Dilawari et al., 2023; Heo, 2023; Kaplon et al., 2023).

##### 2.8.2.6 Disitamab vedotin

Disitamab vedotin (Aidixi®; RemeGen) was approved by the NMPA (National Medical Products Administration of China) in 2021 as a second-line treatment for patients with HER2-expressing, locally advanced or metastatic urothelial carcinoma (mUC) who have previously received Pt-containing chemotherapy (Fu et al., 2022), and approved for patients with HER2-overexpressing locally advanced or metastatic gastric cancer who have received at least two systemic chemotherapy regimens (Deeks, 2021). It delivers HER2+ cancer cell MMAE (DAR equals 4) via a cleavable linker bound to a humanized mAb. Interestingly, Aidixi showed high specific antigenic activity and stronger tumor activity compared to other HER2+-targeted ADCs in preclinical experiments and in animal models (Shi et al., 2022).

##### 2.8.2.7 Cetuximab sarotalocan

Cetuximab sarotalocan (Akalux®; Rakuten Medical) received PMDA (Pharmaceuticals and Medical Devices Agency of Japan) approval in 2020 (Gomes-da-Silva et al., 2020). It is comprised of a chimeric IgG1 mAb specifically targeting epidermal growth factor receptor (EGFR), the triggering of which is involved in cell proliferation, angiogenesis, and invasion/metastasis. The conjugation of Akalux is not with a small molecule, but with a light-activatable near-infrared dye called 700DX (DAR 1.3–3.8) (Fu et al., 2022). In this case, we would also like to consider it as ADC given its mechanism of action against cancer cells. After interaction with ADC-EGFR, this ADC inhibits EGFR signaling pathway and achieves high anticancer effect by laser activation of 700DX dye. In this way, malignant cells are targeted and rapidly eliminated while healthy cells surrounding the tumor mass are spared. It has been approved for the treatment of unresectable locally advanced or recurrent head and neck squamous cell carcinoma (HNSCC) (Kitamura et al., 2020; Omura et al., 2023).

### 2.9 The therapeutic strategy of ADC reveals challenges and limitations and can be combined in combinatorial regimens

#### 2.9.1 Payloads are the main cause of ADCs toxicity

In 2 decades, ADCs have achieved remarkable results in the treatment of hematologic malignancies and solid tumors and represent a valid alternative in the field of clinical oncology. Although they have been designed to release cytotoxic agents by targeting selective cell populations, a striking number of clinical trials have shown that ADCs are not free of adverse effects, sometimes leading to toxicities commonly observed with conventional chemotherapy (Dumontet et al., 2023; Tarantino et al., 2023). In general, a significant proportion of patients suffered various toxicities, sometimes so severe (or fatal) to require dose reduction or interruption, treatment delays, and supportive medications (Dumontet et al., 2023; Tarantino et al., 2023). Each of the blocks that form an ADC can result in significant side effects when these compounds are administered to the human body. Even if the nature of mAbs is responsible for moderate/severe immunogenic side effects, especially in ADCs preparations using murine and chimeric mAb scaffolds (Kim and Kim, 2015), the primary manifestation of a toxicity profile is highly dependent on the type of payload (Zahavi and Weiner, 2020). Most ADCs used in the clinic are loaded with tubulin inhibitors or DNA-interacting agents that exert their cytotoxic effects in the range of nano- or picomolar concentrations and are highly toxic when used in unconjugated form (Mecklenburg, 2018). Unfortunately, because only a very small percentage of ADCs reach their targets and a part of the payload is released prematurely, a significant portion of the dose is virtually free to interact with numerous non-target healthy cells and cause unconventional systemic or local side effects (Dahlgren and Lennernäs, 2020). The out-of-target toxicities are closely related to the linker. Non-cleavable linkers exhibit greater stability in plasma and the ADCs are better tolerated by the body. However, in the majority of ADCs used in the clinic and foremost to the ones use in solid tumors, cleavable linkers are preferred as they have shown more benefits, probably due to the bystander side effect. This out-of-cell toxicity has two sides. On the one hand, it may extend the efficacy of treatment to antigen-negative cells found in the tumor niche, to cells that form the core of the tumor mass being less accessible to the ADC (Jain, 2005; Mahadevan and Von Hoff, 2007; Xiao and Yu, 2021), and to cells that have low/heterogeneous expression of the antigen. On the other hand, due to the non-specific effect of the conventional small drugs, normal cells can also suffer severe damages with potential unpredictable consequences (Donaghy, 2016; Staudacher and Brown, 2017). Other limitations relate to the pharmacokinetic properties of ADCs. Rapid clearance and aggregation represent two important aspects that may negatively impact the therapeutic activity of these compounds (Lucas et al., 2018; Mahmood, 2021; Pettinato, 2021). To solve complications and improve the body compatibility of ADC, the mAb component and linker can be chemically modified by glycosylation or PEGylation (Mahmood, 2021; Edwards et al., 2022). While the former has not been studied in detail within this platform, the latter allows overcoming some drawbacks (Pettinato, 2021). PEGylation involves the addition of polyethylene glycol (PEG) to specific amino acid residues. In general, it has been shown that the use of PEG as a linker can improve the solubility of ADCs and reduce aggregation, improving their pharmacokinetic by enhancing stability and distribution in the body (Verhoef and Anchordoquy, 2013; Mahmood, 2021).

#### 2.9.2 Clinical manifestations of ADCs include major toxicities

As ADCs are developed to limit their exposure to healthy tissues, they are associated with quite manageable toxicities, with nausea, vomiting, diarrhea and fatigue being among the most frequent ones. Unfortunately, the large amount of data provided by several clinical trials also highlights the presence of severe toxicities (grade 3 or higher) that include peripheral neuropathy and hematotoxicity, often dose-limiting (Masters et al., 2018; Professional Committee on Clinical Research of Oncology Drugs et al., 2022). Peripheral neuropathy includes tingling, pain in the extremities, numbness, and rarely muscle weakness and is commonly associated with ADCs carrying cleavable linkers bound to tubulin inhibitors (i.e., all ADCs loaded with MMAE) and Mirvetuximab soravtansine, carrying DM4 (Nguyen et al., 2023). As cleavable linkers are associated to the premature drug release and that these compounds block tubulin polymerization, this common side effect is not unexpected as microtubules are deeply involved in the axonal transport, an essential process to the growth and maturation of neurons (Yogev et al., 2016). Hematologic side effects include anemia, neutropenia, thrombocytopenia, leukopenia and are mainly due to the off-target Fc receptor-mediated uptake of ADCs into immune cells, with neutropenia being the most prominent toxicity in ADC-based monotherapy (Mahalingaiah et al., 2019). Besides, major toxicities responsible for dose limitations are related to drug classes and mainly include hepatotoxicity (for MMAF, DM1, and calicheamicin), skin toxicity (for MMAE and PBD), and ocular toxicity (for MMAF and DM4) (Zhao et al., 2020; Hurwitz et al., 2023). Of note, the mechanism underlying corneal toxicity has not been solved yet but occurred in a relevant percentage of patients treated with Belantamab mafotodin, Trastuzumab emtansine and Mirvetuximab soravtansine, all loaded with tubulin inhibitors (Aschauer et al., 2022; Domínguez-Llamas et al., 2023). Other relevant clinical manifestations include gastrointestinal side effects upon administration of Sacituzumab govitecan and Trastuzumab deruxtecan, two ADC used in the treatment of breast cancer and loaded with Topoisomerase inhibitors and serosal effusion for duocarmycin and PBD, the latter responsible of nephrotic toxicity in Loncastuzimab tesirine preparation (Nguyen et al., 2023). In addition, Trastuzumab emtansine and Trastuzumab deruxtecan, both targeting HER2+ breast cancer, are associated with an increased risk of interstitial lung disease (ILD)/pneumonitis, which must be carefully managed with dose adjustment and supportive care recommendations to avoid fatal outcomes (Ma et al., 2018; Hackshaw et al., 2020). Like other strategies based on small molecules and immunotherapy, ADCs show some challenges that need to be carefully addressed to improve their efficacy and reduce systemic side effects. A variety of approaches is being taken into consideration in clinics to deeply counteract the manifestation of side effects, and many of them focus on an individual basis. Extensive efforts are being made to identify patients who have potentially life-threatening toxicities at an early stage and offer them supportive measures, such as dose and schedule adjustments. The investigation of patients’ history and data on previous treatments, comorbidities, or genetic profiles, including pharmacogenetic analysis to identify SNPs or potential mutations in key genes, will undoubtedly play a critical role in the determination of the best strategy to improve the tolerability and efficacy of ADCs (Lambert and Morris, 2017; Dumontet et al., 2023; Tarantino et al., 2023).

#### 2.9.1 ADCs can be used in combination therapies

In addition to the use of ADCs as monotherapy, recent preclinical and clinical studies have also focused on ADCs combinations with chemo-immunotherapics. Ideally, concomitant administration of ADCs with antiangiogenic agents, i.e., agents that damage neo-blood vessels to facilitate ADC penetration into the tumor niche, or immunomodulatory drugs that promote immune surveillance should amplify the body’s anti-neoplastic response to the tumor mass and its TME, without or with limited severe toxicities and safety issues (Dumontet et al., 2023; Fuentes-Antrás et al., 2023). Regarding ADC combination with chemotherapy, promising data include the combination of Brentuximab vedotin and CHP (cyclophosphamide, doxorubicin, and prednisone) and Polatuzumab vedotin and Rituximab-CHP in CD30^+^ peripheral T cell lymphoma (Herrera et al., 2021) and in DLBCL (Tilly et al., 2022), respectively. Similarly, the combination therapy based on Trastuzumab emtansine and the selective anti-HER2 tyrosine kinase inhibitor (TKI) tucatinib achieved remarkable benefits in metastatic breast cancer (Nader-Marta et al., 2022) and the administration of bevacizumab, the mAb targeting the Vascular-Endothelial Growth Factor (VEGF), with Mirvetuximab soravtansine obtained similar benefits in preclinal models of ovarian cancer (Ponte et al., 2016). Combinatorial approaches using ADCs and immunotherapeutic agents have been recently explored in several types of cancers (Fuentes-Antrás et al., 2023). Immune checkpoint inhibitors (ICIs) are immunotherapy that enhance antineoplastic immune responses by converting exhausted T-cells into activated ones and bypassing the pathways that cause the tumor escape from the immune system (Shiravand et al., 2022; von Arx et al., 2023). ICIs targeting Cytotoxic T-Lymphocyte Antigen 4 (CTLA-4) and the Programmed Cell Death Protein 1/its ligand (PD-1/PD-L1) axis have demonstrated robust clinical activity in several malignancies but only a fraction of patients experience long-term benefits with monotherapy (Wojtukiewicz et al., 2021). From this perspective, an effort could come from ADCs, capable to enhance antitumor immune responses by inducing tumor-specific adaptive immunity through increasing T cell infiltration into the TME, while ICIs revitalize exhausted T cells (Nicolò et al., 2022). Accordingly, encouraging results come from the combination of ICIs and HER2-targeted trastuzumab emtansine in the treatment of PD-L1+, HER2+ advanced breast cancer (Emens et al., 2020).

### 2.10 ADC strategy shows application in non-oncology indications

In recent years, research groups have investigated the use of ADCs in non-oncologic contexts, mainly focusing on the treatment of bacterial infections and autoimmune diseases. Tvilum et al. achieved antimicrobial efficacy by developing an Antibody-Antibiotic Conjugate (AAC), an antibody directed against bacteria conjugated to the antimicrobial molecule mitomycin C, for the treatment of implant-associated biofilm infections caused by *Staphylococcus aureus*, the most common causative agent in prosthetic joint infections (Tvilum et al., 2023). In another study, O-Leary et al. proposed the development of an Antibody-Bactericide Conjugate (ABC), an antibody conjugated to an antimicrobial peptide that exerts its effect by binding to the cell surface of *P. aeruginosa*, providing another interesting example of ADC activity against bacterial infections (O'Leary et al., 2023). Regarding inflammatory diseases, Yasunaga et al. developed an ADC targeting IL-7 receptor (IL-7R) conjugated with MMAE and showed that ADC-mediated immunoregulation of the IL-7R, the upregulation of which is a common mechanism in the pathogenesis of autoimmunity, specifically depleted IL-7R-positive cells in the inflammation site of a mouse model of autoimmune arthritis and abrogated disease progression (Yasunaga et al., 2017). In the same year, Lee et al. demonstrated in a model of rheumatoid arthritis (RA) the immunosuppressive efficacy of TCZ-ALD, an ADC that targets the IL-6 receptor (IL-6R) and is conjugated to the small molecule alendronate. TCZ-ALD blocks the IL-6R activity and macrophages activity in the manifestation of RA symptoms and joint inflammation (Lee et al., 2017). A few years later, Gillard et al. achieved immune reset of disease-causing reactive T cells by a single administration of CD45-ADC and bone marrow transplantation, resulting in significant disease-modifying effect in mouse models of autoimmune disease (Gillard et al., 2020). In addition, a few studies also addressed the use of ADCs in cardiovascular (Lim et al., 2015) and renal diseases (Kvirkvelia et al., 2015), highlighting the potential application of this therapeutic strategy in other pathological conditions.

### 2.11 Challenges and solutions in the manufacturing of ADCs

#### 2.11.1 Process development consists of several steps

The main goal in the manufacture of ADCs by companies is to produce a pure and bioburden-free compound that is safe for the human body for clinical use. Since the preparations of ADC consist of mixtures of mAbs with multiple conjugation sites and small organic molecules, several analytical steps are required in the development of these compounds, and several challenges must be overcome. Overall, the production of ADCs can be divided into three steps: production of the antibody, synthesis of the drug-linker complex, and conjugation to form the final ADC (Hutchinson et al., 2018; Bulger et al., 2023). The conjugation step is of fundamental importance as it determines the therapeutic efficacy of the biomolecule. Conventional conjugation methods based on lysine side-chains or reduced cysteines result in heterogeneous ADC preparations whose safety and therapeutic efficacy are difficult to predict. To overcome this crucial limitation, site-selective conjugation methods have recently been developed. In this way, a known number of drug molecules are constantly bound to selected sites on mAbs, resulting in a more homogeneous mixture with improved batch-to-batch consistency and therapeutic efficacy (Cao et al., 2019; Nejadmoghaddam et al., 2019). After the conjugation step, ADC proceeds to purification and filling into aseptic vials, all following the sterile pipeline of current Good Manufacturing Practices (cGMP). Quality is controlled throughout the development process. Production requires a biological manufacturing environment that allows for the safe handling of sensitive structures, such as various powders and chemicals, purified mAbs, and high potency drugs, to minimize potential pollution and losses that may occur at various stages of production. To ensure product purity and sterility, synthesis and bioconjugation reactions are performed in aseptic rooms and conditions, with regular documentation of instrument accuracy in accordance with cGMP standards. To reduce potential contamination and exposure from the use of hazardous substances, each step of the experimental process is achieved by providing in-depth expertise and specialized equipment to personnel and operators (Hutchinson et al., 2018; Schmidhalter et al., 2019). In addition, the production of ADC usually involves several departments/services and, in this sense, an extremely low temperature supply chain and secure packaging must be ensured to reduce possible variations, damage and leakage during long-term storage and transportation (Ducry, 2012). Considering the complexity and number of processes involved, the production of ADCs is quite laborious, time-consuming and economically expensive. Large capital investments are required to cover a multitude of steps ranging from the design of the experiment, the invention and development of new drugs to product innovation, differentiation, and safety monitoring, all at a cost that must make the finished drug marketable (www.cbo.gov). To overcome these barriers, contract development and manufacturing organizations (CDMOs), companies that provide drug development and manufacturing services to the pharmaceutical industry, are turning to single-use technologies (SUTs) and automated end-to-end systems (Schmidhalter et al., 2019). SUTs are sterile, single-use items made of various plastics, most of which can be used in the same way as their stainless-steel counterparts without the need for sterilization and recycling after use. As a result, these technologies offer significant advantages over traditional reusable systems by reducing the risk of cross-contamination, better ensuring sterility, allowing more flexibility throughout the process and, most importantly, improving cost efficiency and reducing time to market (Schmidhalter et al., 2019). In these terms, continuous improvement in technology and investments by major biopharmaceutical companies in extensive research programs will drive the growth of the ADC market (valued at approximately $7 billion in June 2022 but expected to reach $22.4 billion in 2030 (www.researchandmarkets.com) and increase the number of available ADC-based therapies in new medical areas.

#### 2.11.2 Analytical characterization startegies

Another challenge in the production of ADCs is the evaluation of their biochemical attributes to obtain a safe and effective product. Critical quality attributes of ADCs that must be carefully controlled during the manufacturing process include determination of DAR and distribution of drug, residual non-conjugated species, especially in terms of mAb and payload, and evaluation of size and charge variants in the final preparations. Nowadays, various *in vitro* instruments and techniques are used either alone or in combination to perform comprehensive analytical characterization of ADCs, including spectroscopic, chromatographic, and mass spectrometric methods (Wagh et al., 2018; Liu et al., 2022).

##### 2.11.2.1 Determination of purity

Purity of compound is a fundamental goal in any biopharmaceutical manufacturing process. One of the techniques used to evaluate the purity of ADC preparations is size exclusion chromatography followed by ultraviolet detection (SEC-UV), a method that separates molecules by size and, in some cases, molecular weight, and is used to fractionate large macromolecules such as proteins (e.g., mAbs) or protein complexes (de Mel et al., 2019). In ADC synthesis, separation of macromolecules by size allows purification of the mAb from potential fragments, aggregates, and particles, three examples of undesirable species that affect the efficacy of the final ADC as well as its safety when administered to patients (Jiang et al., 2019). In general, coupling SEC with multi-angle laser light scattering (SEC-MALS) offers some advantages for biopharmaceutical applications. In MALS detection, a laser beam passes through a sample solution containing the target molecule, and depending on the size of the molecules, the intensity of the scattered light is measured at specific angles (Some et al., 2019). Compared to SEC itself, SEC-MALS requires high-purity columns but provides additional information by increasing the sensitivity for detecting impurities in the preparation (Beck et al., 2019). Other techniques for determining the presence of aggregation or fragmentation include dynamic light scattering (DLS), sedimentation velocity analytical ultracentrifugation (SV-AUC) (Ducry, 2012), and capillary electrophoresis followed by sodium dodecyl sulfate analysis (CE-SDS), a valuable method commonly used in the biopharmaceutical industry for mAbs and ADC preparations to determine batch consistency and overall protein purity (Wagner et al., 2020). In addition, various residual species in ADC preparations may pose a potential safety risk to patients and are a critical quality attribute that must be carefully evaluated. The levels of these unconjugated forms can be monitored by measuring the charge on the molecules (Wakanar, 2011). Since the bioconjugation reaction between the mAb and the payload can significantly alter the electrostatic profile of the newly formed ADC, charge-based separation techniques such as ion exchange chromatography (IEC), isoelectric focusing gel electrophoresis (IEF), and capillary isoelectric focusing (cIEF) can provide information on charge heterogeneity, drug distribution pattern, and overall preparation quality (Ducry, 2012). To date, imaged capillary isoelectric focusing (iCIEF) is considered a robust method in biopharmaceutical quality control because it can quantitatively separate samples based on the isoelectric point (pI) of individual variants (Wagh et al., 2018; Abbood, 2023). Therefore, iCIEF can be used to rapidly measure the content of free mAb in an ADC mixture according to the different pI of the mAb and its conjugated form. However, the drawbacks of this assay are that conjugates cannot be distinguished from reaction intermediates and other impurities, and it can only be used for conjugation chemistries that result in significant changes in charge and net pI (Wagh et al., 2018).

##### 2.11.2.2 Determination of DAR and drug distribution

Probably the hottest topic in the ADC manufacturing process is the ability to achieve bioconjugation reactions that lead to the synthesis of a homogeneous mixture and thus a controlled DAR and payload. Various analytical methods have been used to determine these parameters, including ultraviolet/visible spectroscopy (UV/Vis) (Andris et al., 2018), hydrophobic interaction chromatography (HIC) (Andris and Hubbuch, 2020), reversed-phase liquid chromatography (RPLC) (Chen et al., 2019), and mass spectrometry (MS). As for MS, its extended use in characterization of ADCs is discussed in other works (Debaene et al., 2014; Zmolek et al., 2016; Liu and Chen, 2022; Barbero et al., 2023). UV/Vis spectrometry is relatively easy to measure DAR compared to the other techniques, although it requires sufficiently different absorption profiles between the mAb and the payload and a UV/Vis chromophore on the payload (Wagh et al., 2018). Among liquid chromatographic methods, HIC and RPLC are routinely used to measure average DAR and drug distribution (Ouyang, 2013; D’atri et al., 2019). HIC is a method that allows determination of species distribution based on differences in their hydrophobic properties and uniquely preserves the native structures and activity of ADCs. Because the analysis is performed under mild, non-denaturing conditions, ADCs can be studied in their native conformation, which is an advantage because isolation of chromatographically pure species allows their further characterization in subsequent analysis. This method is commonly used to analyze cysteine-conjugated ADCs and other site-specific conjugations but cannot be applied to ADCs obtained by lysine conjugation because the greater heterogeneity of these preparations complicates chromatographic separation (D’atri et al., 2019; Liu and Chen, 2022). Nevertheless, HIC requires a large amount of starting material, shows low efficiency for randomly conjugated ADCs, and cannot separate positional isomers from cysteine-conjugated ADCs or determine the chain, H or L, to which the drug was conjugated (Becker et al., 2020; Fleming, 2020; Liu and Chen, 2022). The RPLC method also separates the components of a mixture according to their hydrophobicity, but this approach requires denaturation of the proteins, resulting in the loss of some information about the distribution of some ADCs and the loss of certain DAR species (Liu and Chen, 2022).

### 2.12 Conclusion and perspectives

**FIGURE 3:**
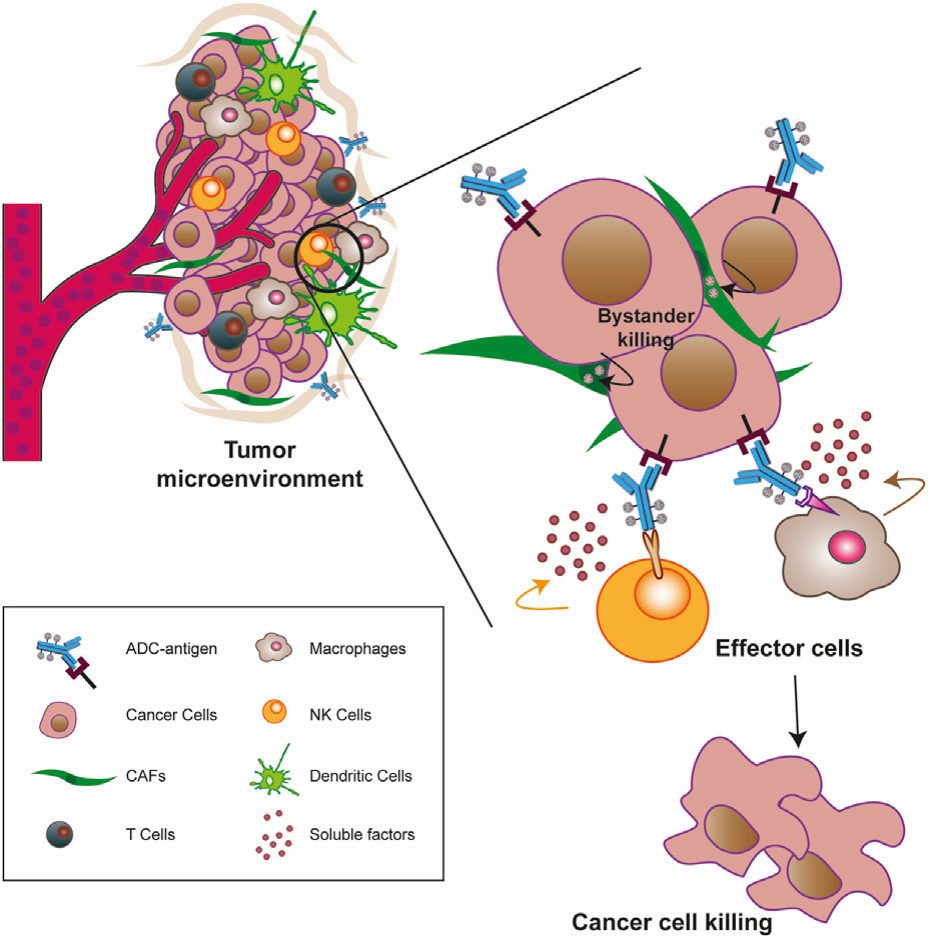
Overview on the tumor-targeting ADCs. Taking advantage of blood circulation, ADCs reach the tumor microenvironment (TME), which is composed by cancer-associated fibroblasts (CAFs) and other cell types and interact with malignant cells that exposed the tumor associated/specific antigen on their surface. In addition to their canonical mechanism of action, ADCs can potentiate their positive response against the tumor mass. To this aim, ADCs hydrophobic payloads may diffuse through the cell membrane inducing the killing of neighborhood antigen-negative cells via bystander killing effect (black arrows) and/or the ADCs antibody Fc fragment may elicit anti-tumor immunity (ADCC, CDC, and ADCP) by engaging immune effector mechanisms, such as complement system, macrophages and NK cells. All together, these mechanisms aim to induce the death of the cellular component of the tumor mass via apoptosis.

Nowadays, ADC represents a solid strategy to treat different types of malignancies. The design of ADCs provides an exceptional opportunity to selectively deliver an effective anti-tumor chemical to the target cell and eliminate it without severe toxic off-target effects. The main advantage of ADC lies in its mechanism of action, as it offers the potential to overcome some major limitations of conventional small molecule-based therapies, such as low therapeutic index and high off-tumor toxicity The possibility of killing neighboring antigen-negative cells, through the bystander effect, in the mass and in the TME and the potential activation of direct and indirect anti-cancer mechanisms via mAb-antigen interaction and immune cell activation, respectively, argue for their use in the clinic (Figure 3). In this context, it is worth noting that the benefits associated with the activity of ADCs when administered as monotherapy may encounter consistent clinical limitations due to the presence of tumor-derived resistance mechanisms and several manageable and few severe adverse effects mainly caused by the payload of the ADC (Fuentes-Antrás et al., 2023). Despite the promising results obtained so far, the ADC technology is still under investigation and has some limitations in terms of its pharmacokinetic properties and biological efficacy in different tumor contexts. To develop a more suitable generation of ADCs, future prospects in this field are based on the optimization of available technologies and especially on the discovery of new methodologies. Accordingly, profound improvements in the validation of newly developed mAbs, in the synthesis of less immunogenic and more stable linkers, and in the discovery of more effective payloads are being actively explored by scientists in this field, and similar advance are also focused on the development of more appropriate formulations as well as on the identification of new target antigens. Given the central role of the TME in solid tumor progression and spread, targeting novel candidates upregulated in stromal cells, blood vessels and most importantly immune cells within the TME could lead to greater inhibition of tumor escape mechanisms and metabolic dysfunctions to achieve long-lasting therapeutic effects. Moreover, combinatorial therapies with different drug classes have already shown synergistic and promising results in the treatment of hematologic and solid tumors by enhancing the anticancer efficacy and therapeutic index of ADCs. Based on these observations, all these efforts are aimed at developing a new generation of ADCs that will undoubtedly show significant improvements in terms of pharmacological properties, therapeutic efficacy and safety in the field of oncology and in other pathological conditions.
